# Back Propagation Neural Network-Based Magnetic Resonance Imaging Image Features in Treating Intestinal Obstruction in Digestive Tract Diseases with Chengqi Decoction

**DOI:** 10.1155/2021/1667024

**Published:** 2021-12-24

**Authors:** Yongfeng Li, Kaina Wang, Li Gao, Xiaojun Lu

**Affiliations:** ^1^Department of Emergency Medical, Xianyang Central Hospital, Xianyang 712000, Shaanxi, China; ^2^Department of Encephalopathy, Xi'an Hospital of Traditional Chinese Medicine, Xi'an 710000, Shaanxi, China; ^3^Department of Pain, Xianyang Central Hospital, Xianyang 712000, Shaanxi, China; ^4^Department of Geriatrics, Xianyang Central Hospital, Xianyang 712000, Shaanxi, China

## Abstract

This study was to explore the adoption effect of magnetic resonance imaging (MRI) image features based on back propagation neural network (BPNN) in evaluating the curative effect of Chengqi Decoction (CD) for intestinal obstruction (ileus), so as to evaluate the clinical adoption value of this algorithm. Ninety patients with ileus were recruited, and the patients were treated with CD and underwent MRI scans of the lower abdomen. A BPNN model was fabricated and applied to segment the MRI images of patients and identify the lesion. As a result, when the overlap step was 16 and the block size was 32 × 32, the running time of the BPNN algorithm was the shortest. The segmentation accuracy was the highest if there were two hidden layer (HL) nodes, reaching 97.3%. The recognition rates of small intestinal stromal tumor (SIST), colon cancer, adhesive ileus, and volvulus of MRI images segmented by the algorithm were 91.5%, 88.33%, 90.3%, and 88.9%, respectively, which were greatly superior to those of manual interpretation (*P* < 0.05). After the intervention of CD, the percentages of patients with ileus that were cured, markedly effective, effective, and ineffective were 65.38%, 23.16%, 5.38%, and 6.08%, respectively. The cure rate after intervention of CD (65.38%) was much higher in contrast to that before intervention (13.25%) (*P* < 0.05). In short, CD showed a good therapeutic effect on ileus and can effectively improve the prognosis of patients. In addition, MRI images based on BPNN showed a good diagnostic effect on ileus, and it was worth applying to clinical diagnosis.

## 1. Introduction

The intestine is an important physiologically active organ in the digestive system. It takes an imperative part in maintaining the homeostasis and regulation of the neuroendocrine and immune system [1]. Intestinal obstruction refers to an acute abdominal disorder in which the contents of the intestine cannot pass through the intestine smoothly due to various pathological changes, which leads to abdominal pain, flatulence, defecation, and stopping of exhaust. Intestinal obstruction is often caused by factors such as irregular diet, excessive fatigue, congestion in the body, clusters of intestinal parasites, and cold, heat, and damp pathogens [2]. Due to different causes, different clinical symptoms are often manifested, such as mild abdominal tenderness, pleural swelling, no defecation, thinning and whitening of the tongue coating, or abdominal swelling, severe pain, hyperintestinal sounds, and mild pleural irritation [3, 4]. Intestinal obstruction belongs to the category of “guange” and “ileus” in Chinese medicine. The intestines are the “home of transmission and transformation” and have the functions of food delivery, digestion, and transfusion. Its physiological characteristics are “purge without hiding,” “moving but not static,” “falling but not rising,” and “actual but not full” [5, 6]. The tongjiang disorder, the obstruction of food worm, and so on, can all lead to the onset of intestinal Qi and blood lumps and blockage, which manifests as pain, vomiting, swelling, and occlusion. Therefore, generalization is the main rule for the treatment of ileus. Traditional Chinese medicine (TCM) often chooses Chengqi Decoction (CD) to treat continuous abdominal distension and abdominal pain caused by ileus [7, 8].

At present, in clinical treatment, routine radiology, ultrasound, computerized tomography (CT), magnetic resonance imaging (MRI), and other imaging techniques are often used to identify ileus, determine the location of obstruction, and determine the cause and severity of obstruction [9]. Through abdominal imaging, it can initially be observed whether there is gas or fluid accumulation in the intestinal tract, the location of obstruction would be determined, and the cause of ileus can be found. The imaging manifestations of ileus are shown as intestinal dilatation, gas and fluid accumulation, uneven gas and liquid planes, short liquid plane, and high gas column in the intestinal cavity. However, it is difficult to reflect the specific conditions of the lesions with ultrasound, CT, and more imaging examinations, and there is a risk of misdiagnosis and missed diagnosis highly depending on the experience of the clinician.

Back propagation neural network (BPNN) is a multilayer feedforward network trained according to the error back propagation (BP) algorithm [10, 11]. The human brain is composed of multiple neurons with certain connections among each other, which becomes the basic element for the brain to process information. The calculation and judgment process in the artificial neural network (ANN) was optimized by imitating the human brain, for the functions that the current computer cannot achieve [12, 13]. BP is widely used in the auxiliary diagnosis and survival analysis of diseases and can be used for disease screening. Medical image classification is a key point of current medical diagnosis and pattern recognition. In medical images, the identification of diseased tissues (ultrasound, CT, MRI, etc.) is realized via image recognition and classification, and information, including the location of the disease, size, and quantity of the disease, is determined [14]. As the texture features of medical images are combined with BPNN and the trained neural network is used for image classification and recognition, the given medical images are effectively and accurately identified and classified, with pixel feature information taken as samples in the training of the neural network [15, 16].

Based on this, a BPNN model was built and applied to the classification and recognition of ileus MRI images in this study, so as to explore the adoption value of BPNN-based MRI impact features in the curative effect evaluation of CD treatment of ileus.

## 2. Materials and Methods

### 2.1. General Data

Ninety ileus patients who were admitted to the hospital from March 2019 to September 2020 were included, including 48 male patients and 42 female patients. The study was approved by the ethics committee of hospital, and the corresponding informed consent was given by all the patients.

The inclusion criteria were determined as follows: patients who were diagnosed as ileus by premenstrual pathology and imaging; patients aged 35–60 years; patients who had not received other medications recently during the enrollment study; and patients who met the diagnostic criteria of TCM syndromes.

The exclusion criteria were given as follows: patients with heart, brain, lung, liver, and other system or organ diseases; patients who were unable to receive treatment; and patients with incomplete clinical data and medical history information.

### 2.2. CD Enema Treatment and the Diagnostic and Therapeutic Criteria

The patients were treated with TCM enema, specifically, and CD enema was used to treat ileus. Rhubarb (20 g), mirabilite (20 g), *Citrus aurantium* (20 g), and *Magnolia officinalis* (20 g) were boiled into 100 mL of decoction and transferred to the infusion bottle, which was connected with the infusion set. The connector was connected to the urinary catheter, which was inserted from the patient's anus to a depth of about 25 cm. The temperature of the decoction was kept at about 37°C, and it was dripped at a rate of 50 drops per minute. After the drug was instilled into the patient's body, it was kept for 2 hours. In addition, on the basis of TCM prescription gavage treatment, gastrointestinal decompression management, electrolyte maintenance, and nutritional intervention were implemented for patients.

The curative effect was evaluated according to the criteria based on Intestinal Obstruction Diagnosis and Therapeutics. “Cured” meant that the patient's symptoms disappeared and there was no gas-liquid level in the intestine; “markedly effective” meant that there was a small amount scattered on the gas-liquid level; “effective” meant that all symptoms were greatly reduced and the intestine was scattered on the gas-liquid plane; and “invalid” meant that there was no obvious improvement in all symptoms.

### 2.3. MRI Scan of the Patient's Abdomen

The patients included in the study were examined by an MRI scan, using the magnetic resonance instrument. The gradient magnetic field was controlled at 40 mt/m, and the switching rate was set at 200 mt/m*∗*s. The coil was the thoracic and abdomen soft coil or body coil. The CorGRE sequence was scanned routinely, and the oblique Obl GRE of Axi GRE, Axi Double IR FSE, and parallel aortic arch (scanning line passes through the ascending aorta and descending aorta) were scanned. On this basis, MRI middle and lower abdominal plain scan and intestinal water imaging were performed for the patient. Specifically, the half‐Fourier acquisition single‐shot turbo‐spin‐echo (HASTE) sequence and Turbo-Spin-Echo (TSE) sequence were adopted for T2-weighted imaging (T2WI) coronal scanning and the true fast imaging with steady-state precession (True-FISP) sequence coronal and transverse surface scan. In addition, the contrast agent directly used the fluid in the patient's intestinal cavity, and the scanning range mainly included the position of the xiphoid process to the pubic bone. The scanned MRI images of the patient were interpreted by two attending doctors or imaging doctors with rich clinical experience, and the specific location, type, and maximum diameter of the lesion were judged by MRI.

### 2.4. Image Segmentation Based on BPNN

**Figure 1:**
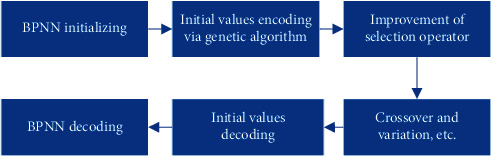
Formation process of BPNN.

BPNN is composed of three layers of feedforward network, namely, the input layer, hidden layer (HL), and output layer. An optimized BPNN model based on improved genetic algorithm was introduced in this study, and its formation process is shown in Figure 1.

The improvement of the selection operator in the genetic algorithm was based on the sorting method, and the image features were grouped according to the degree of fitness. Thereby, new individuals that met the characteristics of the species were generated, and a Gaussian mixture model was formed. The n-order Gaussian mixture model function is shown as follows:

(1) $$\begin{array}{c} p\frac{a}{\emptyset }=\sum_{x=1}^{n}b_{x}p_{x}\left(\frac{a}{\emptyset_{x}}\right), \end{array}$$

where *p*_*x*_(*a*/∅_*x*_) represents the probability density function of the *x*-th branch, and it was expressed as follows:

(2) $$\begin{array}{c} p_{x}\frac{a}{\emptyset_{x}}=\frac{1}{{\left(2\pi \right)}^{2/d}{\left|\sum l\right|}^{1/2}}\times \exp\left(\frac{1}{2}{\left(a-\mu_{x}\right)}^{S}\overset{-1}{\underset{l}{\sum }}\left(a-\mu_{x}\right)\right). \end{array}$$

In this equation, ∅_*x*_=(*μ*_*x*_^*s*^, ∑*x*)^*S*^. *μ*_*x*_ is the expectation of the *x*-th branch, ∑*x* is the d-order covariance matrix of the *x*-th branch, and *n* represents the number of branches of the Gaussian mixture model.

**Figure 2:**
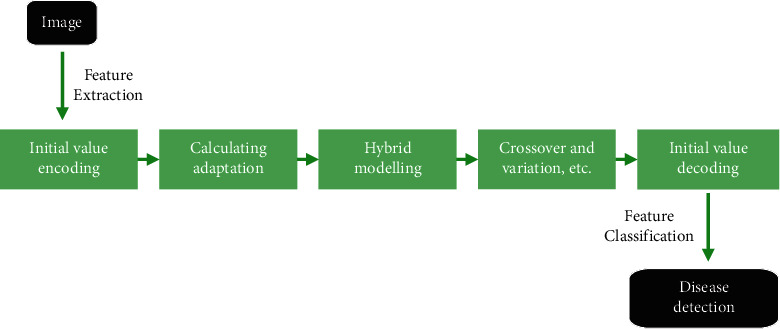
Classification procedure of the optimized BPNN model under improved genetic algorithm.

By improving the genetic algorithm, the maximum fitness value of each generation of the species was calculated. When the maximum fitness value did no longer change significantly or reached the maximum evolution algebra of the species, the calculation was stopped. The optimal weight and threshold were obtained through decoding and then were assigned to the neural network for training. The error was output after calculation, and when the error reached the preprogrammed precision or the predetermined number of training times, the training was ended. The optimization and establishment BPNN were completed, and the specific process is shown in Figure 2.

### 2.5. Effect Evaluation

BPNN algorithm was experimentally verified and used for the segmentation of abdominal MRI images of ileus patients. Based on the segmentation technology of graph cutting, the image was mapped into a network graph, and the segmentation problem was transformed into different operations on the graph. The maximum flow/minimum cutting theorem was adopted to realize the segmentation of the image. In addition, the edge detection algorithm and threshold segmentation algorithm were introduced and compared to analyze the adoption value of BPNN in ileus MRI image segmentation.

### 2.6. Statistical Analysis

The data were processed and analyzed by SPSS 19.0, and the measurement data were expressed in the form (*x* ± *s*). The percentage (%) referred to how many count data were expressed. Analysis of variance (ANOVA) was used in pairwise comparison. *P* < 0.05 indicated that the difference was statistically considerable.

## 3. Results

### 3.1. Running Time of BPNN Algorithm

**Figure 3:**
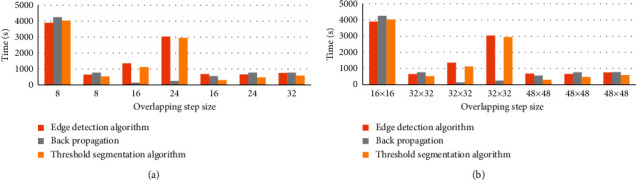
Running time of different algorithms on image reconstruction. (a), (b) The running time of different overlap steps and block sizes.

Figure 3 shows the running time of the edge detection algorithm, threshold segmentation algorithm, and BPNN algorithm for MRI image reconstruction. Figures 3(a) and 3(b) illustrate the operating times under different block divisions and different overlap step parameter settings, respectively. When the overlap step was 8 and the block size was 32 × 32 and when the overlap step was 16 and the block size was 48 × 48, the running time of the edge detection algorithm and the threshold segmentation algorithm was the shortest. In addition, when the overlap step was 16 and the block size was 32 × 32, the running time of the BPNN algorithm was the shortest.

### 3.2. Diagnostic Accuracy of Different HL Nodes

**Figure 4:**
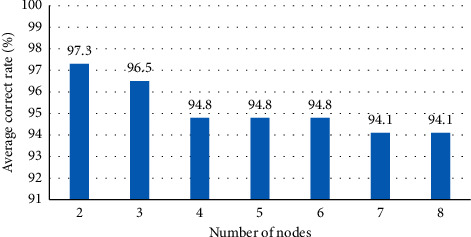
The average correct rate of different HL nodes.

BPNN has a forward network with three layers or more. Any continuous function in the closed interval can be approximated by a BPNN with single HL. Therefore, a simple cross-validation method was used to test the number 2–8 of possible HL nodes, so as to study the influence of the neural network structure of different HL node number models on the accuracy of MRI image segmentation. As shown in Figure 4, when the number of HL nodes was 2, 3, 4, 5, 6, 7, and 8, the corresponding segmentation accuracy was 97.3%, 96.5%, 94.8%, 94.8%, and 94.1%, respectively. When the number of HL nodes was 2, the segmentation accuracy was the highest, which was 97.3%. Therefore, the number of BPNN HL nodes was set to 2 in this study.

### 3.3. MRI Image of Patients with Ileus

**Figure 5:**
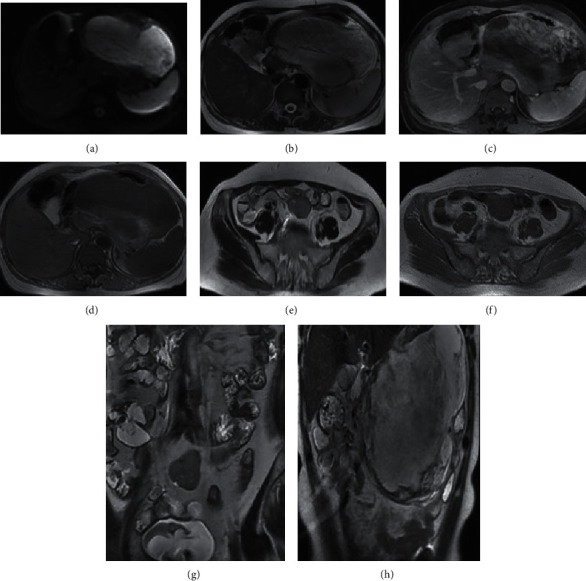
MRI image of patients with ileus. (a–d) Coronal MRI images; (e, f) transverse MRI images; and (g, h) aberrational MRI images.

The lower abdominal MRI images of a 46-year-old female patient with ileus are shown in Figure 5. The MRI images of transverse, coronal, and aberrations showed that the proximal intestine of the obstruction was dilated and air-liquid planes were visible. In addition, the soft tissue massed at the obstruction site, the intestinal wall was irregularly thickened, and the intestinal lumen was narrow. The obstruction site on T1WI was similar to the muscle model, showing low signal, and it showed high signal on T2WI.

### 3.4. The Segmentation Effect of the Ileus MRI Image

**Figure 6:**
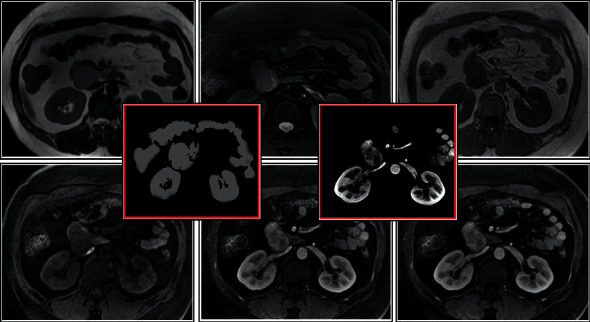
The segmentation effect of the ileus MRI image.

BPNN firstly forwards the input signal to the HL node, then passes the output information of the HL node to the output node after the action, and finally, outputs the result. The input image is trained by a neural network to complete the classification task, the classification result is converted from a one-dimensional vector array into an image matrix form, and the segmentation result is displayed, thereby realizing image recognition and segmentation. As given in Figure 6, BPNN showed high accuracy in identifying and segmenting lesions in MRI images of ileus patients.

### 3.5. Diagnosis Accuracy of Ileus

**Figure 7:**
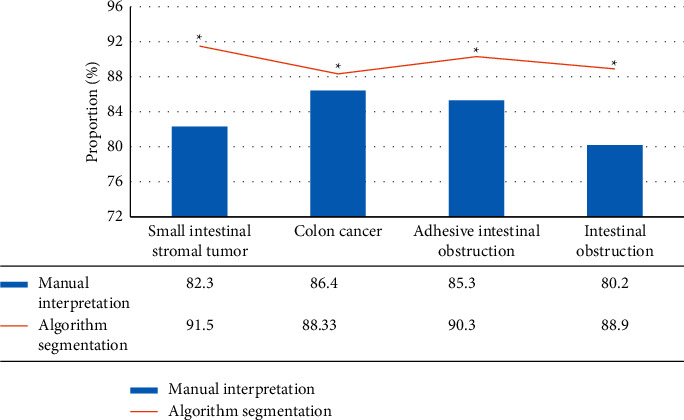
Diagnosis accuracy of ileus. ^*∗*^There was a statistically significant difference compared to the algorithm segmentation (*P* < 0.05).

Figure 7 showed the recognition accuracy of different types of ileus after the MRI image was segmented by the clinician's manual interpretation and algorithms. It included the malignant ileus (SIST and colon cancer), adhesive ileus, and volvulus. It illustrated that the recognition rates of SIST, colon cancer, adhesive ileus, and volvulus of MRI images segmented by the algorithm were 91.5%, 88.33%, 90.3%, and 88.9%, respectively, which were dramatically superior to the recognition rates of manual interpretation (*P* < 0.05).

### 3.6. Improvement on the Condition of Patients before and after Intervention

**Figure 8:**
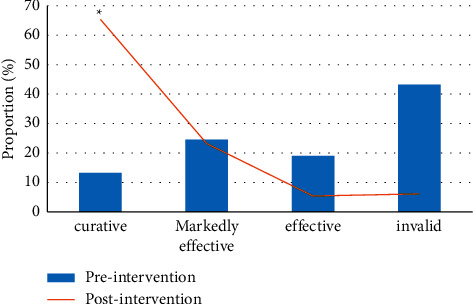
Improvement on the condition of patients before and after intervention. ^*∗*^The difference was statistically significant compared to that after intervention (*P* < 0.05).

Figure 8 showed the improvement of patients' ileus before and after CD intervention. The figure revealed that, after CD intervention, the proportions of ileus patients being cured, markedly effective, effective, and ineffective were 65.38%, 23.16%, 5.38%, and 6.08%, respectively. The cure rate of CD after intervention (65.38%) was greatly superior to that before intervention (13.25%) (*P* < 0.05).

## 4. Discussion

Intestinal obstruction, also called ileus, refers to an acute abdominal disorder in which the contents of the intestine cannot pass through the intestine smoothly due to various pathological changes. It will lead to abdominal pain, flatulence, defecation, and stopping of exhaust. Intestinal obstruction is often caused by factors such as irregular diet, excessive fatigue, congestion in the body, clusters of intestinal parasites, and cold, heat, and damp pathogens. The diagnosis of ileus mainly relies on inspection, auscultation, palpation, and abdominal imaging (CT and MRI). Through abdominal imaging, it can be known initially whether there is gas or fluid accumulation in the intestinal tract, where the obstruction occurs, and the cause of ileus. The imaging manifestations of ileus are intestinal dilatation, gas and fluid accumulation, uneven gas and liquid planes, short liquid plane, and high gas column in the intestinal cavity [17, 18]. The liquid planes are arranged in a discontinuous stepped shape with each other. This syndrome is a characteristic manifestation of simple urban obstruction. The lying film shows the continuous expansion of the intestinal cavity. At this time, the degree of expansion of the intestinal cavity and the mucosal structure of the intestinal cavity can be clearly observed. Smooth tends to bend closer together. There are many methods for MRI image segmentations, including manual, semiautomatic, and fully automatic segmentation [19, 20]. Among them, manual segmentation is to directly outline the boundary with a pen on the image, and the segmentation result is completely determined by the practical experience of the segmenter, so the reliability is low. The semiautomatic segmentation and automatic segmentation methods reduce the influence of subjective factors and are fast and accurate [21]. In addition, the automatic segmentation method is completely computer-completed and shows high adaptability to lesions with irregular borders and large individual differences in shape [22, 23]. Deep learning algorithms such as BPNN can complete the classification task by training the input image and convert the classification result from a one-dimensional vector array into an image matrix form. Then, the segmentation result is displayed; thereby, image recognition and segmentation are realized. It plays a key role in the recognition and segmentation of lesions shown in the image, and the segmentation effect is ideal [24].

BPNN was adopted for the segmentation of abdominal MRI images of patients with ileus. Based on the segmentation technology of graph cutting, the image was mapped into a network graph, and the segmentation problem was transformed into different operations on the graph. The maximum flow/minimum cutting theorem was adopted to realize the segmentation of the image. In addition, the edge detection algorithm and threshold segmentation algorithm were introduced and compared to analyze the adoption value of BPNN in ileus MRI image segmentation. The segmentation accuracy was 97.3%, 96.5%, 94.8%, 94.8%, and 94.1%, respectively, if the number of HL nodes in the algorithm model was 2, 3, 4, 5, 6, 7, and 8. When the number of HL nodes was 2, the segmentation accuracy was the highest, which was 97.3%. Therefore, the number of HL nodes of the BPNN was set to 2 in this study. The recognition rates of small intestinal stromal tumors, colon cancer, adhesive ileus, and volvulus of MRI images segmented by the algorithm were 91.5%, 88.33%, 90.3%, and 88.9%, respectively, which were dramatically higher in contrast to the recognition rates of manual interpretation (*P* < 0.05). It suggested that the algorithm shows a better segmentation effect on MRI images of ileus. In addition, after the intervention of the TCM CD for the patients, it was found that the cure rate (65.38%) after the intervention of the CD was higher greatly than that before the intervention (13.25%) (*P* < 0.05). Such results were similar to those of the study of Deane et al. (2018) [25]. It indicated that CD showed a better therapeutic effect on ileus and can effectively improve the prognosis.

## 5. Conclusions

BPNN algorithm was employed for the segmentation of ileus patients' abdominal MRI images. Based on the segmentation technology of graph cutting, the image was mapped into a network graph, and the segmentation was transformed into different operations on the graph. The maximum flow/minimum cutting theorem was adopted to realize the segmentation, and then, the adoption value of MRI image features under BPNN was explored in evaluating the curative effect of CD in the treatment of ileus. It was found that CD showed a better curative effect on ileus and improved the prognosis. MRI images based on BPNN showed a good diagnostic effect on ileus, and it was worth applying to clinical diagnosis. However, it failed to compare with other intelligent algorithms, and the representativeness was low. Therefore, it would make improvements and optimizations in this aspect in subsequent experiments, and further analysis would be performed to analyze the adoption value of BPNN-based MRI image features in evaluating the curative of CD treatment ileus. In short, this study provided a reference for the adoption of BPNN and other intelligent algorithms in medical imaging.

## Data Availability

The data used to support the findings of this study are available from the corresponding author upon request.
